# Editors’ Book Table

**Published:** 1872-08

**Authors:** 


### Editors' Book Table.

[NOTE. — All works reviewed in the columns of the CHICAGO MEDICAL JOURNAL may be found in the extensive stock of W. B. KEEN, COOKE & CO., whose catalogue of Medical Books will be sent to any address upon request.]

BOOKS RECEIVED.

*Aural Catarrh and Curable Deafness.* By PETER ALLEN, M.D., Aural Surgeon to St. Mary's Hospital, etc., etc. For sale by W. B. Keen, Cooke & Co., 408 Wabash Ave., Chicago.

The student of medicine, as well as the practitioner, will find benefit in the perusal of this little work. The anatomy and physiology of the structures of the middle ear, and the symptoms and pathology of its diseases, are plainly discussed.

There is scarcely any class of diseases of which good practitioners are so ignorant, as of this. Any work which presents any portion of aural science in a clear light, is a valuable addition to our literature. There is the less excuse for this ignorance, since aural literature is comparatively limited in extent.

This work, although nominally devoted to a very prevalent disease, aural catarrh—catarrhal deafness—contains many valuable hints regarding aural science generally. We most heartily commend the work to our readers.

We would, however, be more pleased, if, in the author's report of cases, there were less apparent attempts to lead the reader to regard his skill as superior to that of other specialists.

*A System of Surgery: Pathological, Diagnostic, Therapeutic and Operative.* By SAMUEL D. GROSS, M.D., LL.D., D.C.L. Oxon., Professor of Surgery in the Jefferson Medical College of Philadelphia, etc., etc. Illustrated by upwards of Fourteen Hundred Engravings. Fifth Edition, Greatly Enlarged and Thoroughly Revised. In Two Volumes. Philadelphia: Henry C. Lea. 1872. Pp. 1098 and 1170.

The present edition of this magnificent monument of surgical erudition, experience and skill, is brought out in the highest style of that typographical and general excellence which distinguish the eminent publishing house from which it emanates. In its general features it is too well known to need introduction to our readers. Beyond the most of treatises, it discusses the great Principles of Surgery, without an exact and adequate knowledge of which the mere operator sinks to the level of a mere mechanical artisan. Large attention is also paid to the discrimination of diseases, with an elaborate chapter on general diagnosis.

The work, as a whole, is the embodiment of the matured knowledge and experience of a ripe age, after a long life of earnest study, acute observation, and vast opportunities. Over five years have been devoted to revising, and, indeed, re-writing the work. Most heartily do we commend it to the medical public.

*A Year-Book of Therapeutics, Pharmacy and Allied Sciences.* Edited by HORATIO C. WOOD, Jr., M.D., Professor of Medical Botany, University of Pennsylvania; Physician and Lecturer on Clinical Medicine to the Philadelphia Hospital, etc., etc. New York: William Wood & Company. 1872. 8 vo. pp. 360.

This is given in five parts, including Therapeutics, Materia Medica, Toxicology, Prescriptions and Formula, and General Receipts. It is one of the most convenient and perfect of the annual compendiums we have had the privilege of looking over.

PAMPHLETS.

*Archives of Ophthalmology and Otology.* Edited and published simultaneously in English and German, by Prof. H. KNAPP, M.D., in New York, and Prof. S. MOOS, M.D., in Heidelburg. Volume II, No. 2. New York: William Wood & Co. 8 vo. Pp. 316. With numerous plain and colored illustrations.

*Modern Medicine: Its Status in Modern Society.* By HOMER O. HITCHCOCK, A.M., M.D., Kalamazoo, Michigan. The President's Address before the State Medical Society. Delivered at Grand Rapids, Michigan, June 12th, 1872.

*Season of* 1872.—*Warm Springs of Bath County, Virginia.* Pamphlets to be had of Coleman & Rogers, Baltimore, Md.; Purcell, Ladd & Co., Richmond, Va.; or Eubank, Reynolds & Co., at the Springs.

*Annual Address, Delivered before the Medical Association of Central New York, June 18th,* 1872, by the President, B. L. HOVEY, M.D., of Rochester; and An Essay on Asiatic Cholera, by W. S. ELY, M.D., of Rochester.

*Professional Ethics.—The Annual Address before the Minnesota State Medical Society.* By FRANKLIN STAPLES, M.D., President of the Society. Reprinted from the Transactions of the Society, 1872.

*New Remedies.* A Quarterly Retrospect of Therapeutics, Pharmacy and Allied Subjects. Edited by HORATIO C. WOOD, Jr., M.D., Prof., etc. New York: William Wood & Co., Publishers. July, 1872. Vol. 2, No. 1. $2 per annum.

*The Physician's Monitor for* 1872. A Concise Repertory of Useful Information for the Medical Practitioner; containing Recent Discoveries in the Medical World, and New Remedies in Therapeutics and Allied Subjects; Original, and Selected from the most Eminent Authorities, etc., etc. Vol. 1, No. 2. W. A. Townshend, Publisher and Bookseller, 177 Broadway, New York. Price, 25 cents each.

